# The Diagnostic Value of Human Neutrophilic Peptides 1-3 in Acute Pediatric Febrile Illness

**DOI:** 10.3390/jcm12206514

**Published:** 2023-10-13

**Authors:** Eiass Kassem, Maanit Shapira, Miral Sussan, Loay Mahamid, Naama Amsalem, Rami Abu Fanne

**Affiliations:** 1 Department of Pediatrics, Hillel Yaffe Medical Center, Affiliated with the Rappaport Faculty of Medicine, Technion Institute of Technology, Hadera 3810101, Israel; eiaska@clalit.org.il (E.K.); msussan@tzmc.gov.il (M.S.); louis.m@meuhedet.co.il (L.M.); 2 Laboratory Division, Hillel Yaffe Medical Center, Affiliated with the Rappaport Faculty of Medicine, Technion Institute of Technology, Hadera 3810101, Israel; maanits@hymc.gov.il; 3 Department of Cardiology, Hillel Yaffe Medical Center, Affiliated with Rappaport Faculty of Medicine, Technion-Israel Institute of Technology, Haifa 3200003, Israel; naamaa@hy.health.gov.il

**Keywords:** fever, neutrophilic peptide 1-3, viral, misdiagnosis

## Abstract

**Background:** It is prudent to develop biomarkers that enhance the differentiation between viral and bacterial infection in order to support expeditious and judicious antimicrobial implementation in emergency department admissions. Human neutrophilic peptides 1-3 (HNP1-3) are the major neutrophilic peptides with potent antimicrobial activity. **Methods:** We tested the performance of the plasma HNP1-3 test in a prospective observational cohort of children admitted to the emergency department for fever. We validated this test with traditionally used biomarkers and final diagnoses. An expert panel reviewed the patient’s data and gave a final diagnosis. The final diagnosis was classified as definite, probable, or possible. **Results:** A total of 111 children (98 with fever and 13 control) were recruited: 55% male, mean age 6.3 years. Plasma HNP1-3 levels were higher with bacterial infections: 10,428 (5789–14,866) vs. 7352 (3762–10,672) pg/mL, *p* = 0.007. HNP1-3 were negatively correlated with age: r = −0.207, *p* = 0.029. Of the different categorical variables tested, only c-reactive protein (CRP) (≥42.3 mg/dL), neutrophil count (≥10.2), and age (odds ratio = 1.185, *p* = 0.013 and 95%CI = 1.037–1.354) had significant diagnostic capability for bacterial disease prediction. **Conclusions:** Due to its low diagnostic value in febrile patients, the HNP1-3 value is not currently recommended to support pathogen differentiation in children in an emergency setting. Further studies are needed to support its clinical use.

## 1. Introduction

Fever is the leading complaint responsible for roughly one-third of all pediatric office visits. In general, children have an average of 3–6 febrile insults each year. Moreover, 20–40% of parents seek medical help for childhood febrile illness [1,2,3]. A comprehensive screening study of children’s admissions to the emergency room (ER) showed fever as the number one complaint (22.1%), with 10.3% diagnosed with upper respiratory tract infection and 10% sustaining diarrhea [4,5]. Overall, most of the febrile admissions to the ER are related to a self-limited illness, while 5–10% are diagnosed as bacterial infections including pneumonia, UTI, meningitis, sepsis, or joint infection [6,7,8,9]. The differentiation between bacterial and viral infections is frequently challenging due to similar clinical presentations. Concurrently, prompt diagnosis/differentiation is critical as early antibiotic administration is of major importance. Delaying necessary antibiotic treatment may have life-threatening consequences [10]. Misdiagnosis or delayed diagnosis leads to inappropriate antibiotic administration: under-prescription in bacterial infection and unnecessary treatment in viral cases. The implications of the previous two scenarios are direct life threat and the emergence of new drug resistance, respectively. The early application of antibiotics in febrile children relies mainly on clinical judgment, supported by fast laboratory tests, e.g., urinalysis and/or complete blood count (CBC). The gold-standard test for establishing a diagnosis of bacterial infection is a positive culture from a sterile compartment, including blood, urine, or cerebrospinal fluid (CSF). Nevertheless, a final culture result takes more than 24–48 h, making it ineffective in the acute setting. In this regard, several clinical scoring systems have been suggested as helpful in predicting bacterial vs. viral infection in the ER [11,12,13]; however, most have proved clinically disappointing because they were validated for infants or were evaluated in small studies that could not be generalized to all children. One study tested the accuracy of meticulous clinical symptoms and signs evaluation to predict bacterial infection, proving superior results [14]. Another group tested the efficacy of a combined blood test (ImmunoXpert) incorporating tumor necrosis factor (TNF), CRP, and interferonγ (INFγ) against classical markers like CRP alone, CBC, or procalcitonin for confirming bacterial etiology in ER-admitted children. The ImmunoXpert was superior to the classical tests, reporting 93.8% sensitivity and 89.9% specificity for bacterial infection, with 11.7% of patients reporting non-diagnostic results [15].

Acknowledging that host cellular responses to bacterial and viral infections are distinct—being chiefly neutrophilic or lymphocytic, respectively—it is widely agreed that the distinctive cellular host responses could represent prime targets as diagnostic indicators. The latter could be assessed quantitatively (absolute cell count) or qualitatively, e.g., detected at the protein level.

Neutrophils are the primary cells recruited against bacterial infections; the diagnostic yield of a neutrophil count has been previously described [16]. HNP1-3 are the largest group of antimicrobial peptides expressed predominantly in neutrophils and epithelial cells [17]. They play a key role in innate immunity against invading microbes. They constitute >5% of the total protein stored in azurophilic granules. These peptides are relatively small, ranging approximately from 12 to 50 amino acid residues and having a molecular weight of 3–5 kD. HNP1-3 have six conserved cysteine residues stabilized by three or four conserved intramolecular disulfide bonds. These peptides are cationic with at least two positive charges (lysine and arginine residues) [18,19,20]. HNP1-3 exhibit efficient bactericidal activity because they are involved in killing microbial pathogens by disrupting bacterial membranes; HNP1-3 are attracted to the negatively charged microbial membranes by their cationic charge. Once attached, their hydrophobic structure enables them to establish transmembrane pores and disrupt membranes, eventually leading to ion efflux of bacterial cell contents and death [21]. The normal plasma concentration of HNP1-3 is in the nanomolar range (<15 nM in healthy individuals), with a marked surge (around 50 μM) secondary to neutrophil degranulation measured during acute bacterial processes. HNP1–3 molecules are hydrophobic, protease-resistant, and cleared rapidly (t^1/2^ of 9.7 min) from the circulation without being excreted in the urine or feces [22]. HNP1-3 represent the footprint of active neutrophils in the blood and other body compartments. In this study, we tested whether a single plasma test for HNP1-3 would be useful to differentiate between viral and bacterial infections in an ER setting.

## 2. Methods

### 2.1. Subjects

This was a prospective cohort study carried out at Hillel Yaffe Medical Center (Hadera, Israel). Over a period of 6 months from April to October 2019, all consecutive patients aged ≤18 years and ≥3 months with a fever ≥38 °C who were admitted to the emergency department with symptoms/signs lasting a maximum of 7 days were prospectively included in the study. The control group included children in the same age range who were admitted to the pediatric clinic with a body temperature <38 °C and with no history of chronic inflammatory disease. We excluded children with the following: antibiotic treatment at admission, inflammatory/infectious disease in the last 3 weeks, chronic immunodeficient status, known cancer, chronic anti-inflammatory medication, or any chronic inflammatory disease. The study was approved by the Hillel Yaffe Medical Center Institutional Review Board (approval ID 0003–19-HYMC). The study was conducted according to the guidelines and recommendations of Good Clinical Practice and the Declaration of Helsinki. Written informed consent was obtained from each participant or legal guardian, as applicable.

### 2.2. Definition of Bacterial Infection

Patient infection status was defined according to the International Sepsis Definition Conference (ISDC) 2001 [23].

- Definite infection—patients with definite viral/bacterial infection established on clinical and microbiological criteria.
- Probable infection—patients with clinical manifestation of infection plus radiological evidence without microbiological confirmation.
- Possible infection—patients with clinical features of infection without established microbiological or radiological confirmation.

### 2.3. Data Collection and HNP1-3 Measurement

At admission, a plasma blood sample was collected from each patient into tubes with EDTA. Plasma HNP1-3 levels were quantified by ELISA using a commercial human HNP1-3 ELISA Kit (HNP1-3; HyCult Biotechnology, Uden, The Netherlands, Kit#-HK317), with the measurable concentration ranging from 156 to 10,000 pg/mL. Briefly, plasma samples were stored at −80 °C (after being separated by centrifugation at 5000 rpm for 10 min at room temperature) until the HNP1-3 levels were determined within a period not exceeding 3 months.

In parallel, the patients’ characteristics and clinical details were prospectively obtained from reviewing their clinical notes at discharge. The data collected included the patients’ age, gender, and clinical signs of active infection (heart rate, respiratory rate, blood pressure, temperature, and oxygen saturation). In addition, we reviewed different biochemical determinants (e.g., blood count, CRP level, and glucose), microbiology results (e.g., urine culture, blood culture, nasal swab, etc.), and radiological tests. All radiological findings were confirmed by radiologists. The ImmunoXpert kit was used pending kit availability, and the results were included.

### 2.4. Reference Standard: Expert Panel Adjudication and Predetermined Criteria

A panel made up of 3 expert pediatricians reviewed the medical records of each patient. The panel members independently assigned 1 of the following diagnoses: (1) definite bacterial/viral infection, (2) probable bacterial/viral infection, or (3) possible bacterial/viral infection.

The diagnosis was based on a review of all available patient data collected during the hospital stay (or received after discharge, e.g., cultures/PCR/serology results) including but not limited to medical history; complete blood count; CRP levels; radiological tests; blood, urine, or cerebrospinal fluid culture results; and polymerase chain reaction analysis of nasal swabs (when available).

### 2.5. Statistical Analysis

Descriptive statistics in terms of mean, standard deviation, and percentage were calculated for the whole parameters in the study. The normal distribution of the continuous parameters was tested with the Kolmogorov–Smirnov test. As the HNP1-3 parameter was not normally distributed, we used the Mann–Whitney U test and the Kruskal–Wallis test with an adjustment for multiple comparisons of differences between groups. In addition, we divided the HNP1-3 parameter into four quarters. The differences between those four groups and several independent parameters were demonstrated with the Pearson chi-square and Kruskal–Wallis tests. The Pearson correlation was used to test the relationships between HNP1-3 and several independent parameters (age, BMI, CRP, HbA1c%, cholesterol, TG, HDL, and LDL). A univariate analysis model was constructed to predict death, PCI, unstable angina, CVA, and MI by several independent parameters. Also, we used a logistic regression model with stepwise methods to predict the diagnosis of a bacterial infection with an odds ratio and 95%CI. The ROC (receiver operating characteristic) model was used to find the best classification of continuous parameters to predict a diagnosis of bacterial infection (*p* < 0.05 was considered to be significant). The SPSS program, version 28, was used for all statistical analyses.

## 3. Results

A total of 98 pediatric patients (mean age 6.3 years, 55% male) and 13 healthy controls were recruited. The characteristics of the controls and patients in each group are presented in Table 1. All study participants were stable at enrollment. The patients’ diagnoses assigned at discharge are presented in Table 2. The ultimate diagnoses given by the expert panel are presented in Table 3; in the bacterial group, 31.7% of the cases had a definite diagnosis, 53.7% were classified as probable, and 14.6% were labeled as possible. Among the viral group, 54.4% of the cases were classified as definite, while 45.6% were possible. In the bacterial group, the most common diagnosis was pneumonia (53.7%), while upper respiratory infection prevailed in the viral group (63.2%). Among the definite diagnosis subgroup, the most isolated agent in the bacterial group was *E. coli* (31%), while *H. influenza type A* (55%) predominated in the viral group. The detailed distribution of pathogens in both groups is presented in Table 4.

### 3.1. Performance of the HNP1-3 Test

The levels of HNP1-3 were the lowest in the control group, 1975 (530–3715) pg/mL. However, HNP1-3 levels were significantly higher in children with bacterial as compared to viral infections: median (IQR1-3) levels of HNP1-3 were 10,428 (5789–14,866) versus 7352 (3762–10,672) pg/mL (*p* = 0.007). CRP exhibited a similar pattern, with median levels significantly lower in children with a viral infection as compared to a bacterial infection: 20.5 (4.6–42.2) versus 107 (45–215) mg/L (*p* <0.001). Table 5 clearly illustrates a similar pattern of WBC, neutrophils, and glucose levels.

We next tested HNP1-3 levels according to the different levels of diagnosis in each group (Table 6).

The HNP1-3 assay exhibited the most pronounced correlation (*p* = 0.001) in definite or probable bacterial infections compared to definite viral infections (which composed 68% of the total study group) with median levels of 10,840 (6532–14,866) pg/mL versus 6737 (2457–8005) pg/mL, respectively.

**Figure 1:**
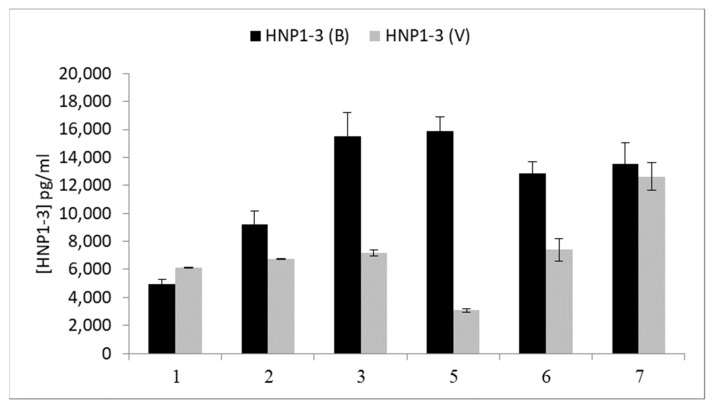
HNP1-3 levels according to the time elapsed from the first symptoms to HNP1-3 blood sampling. B = bacteria; V = viral.

We next tested the median HNP1-3 values in both groups in relation to the time elapsed from the first symptoms’ onset to blood sampling/admission (Figure 1). One can appreciate a no-difference result for HNP1-3 testing on the first day. From days 2 to 6, the HNP1-3 tests were higher in bacterial diagnosis, until declining again on day 7.

We then evaluated the in-hospital occurrence of antibiotic misuse as a result of inappropriate initial diagnosis; in the viral group, 21% of the cases were initially treated with antibiotics that were withdrawn after a median of 3 (2–5) days due to a presumptive alternative viral diagnosis (eight cases of definitive and four cases of possible viral diagnosis). Of note, the ImmunoXpert test was used in 50% of these cases and gave the right diagnosis in only 50% of the tests. The HNP1-3 levels in this group were relatively low, 6139 (4937–8393) pg/mL. In the bacterial group, there were five cases (12.2%) that were initially treated as viral infections and, eventually, covered with antibiotics (two cases of definitive, two cases of probable, and one case with a possible diagnosis). Again, the ImmunoXpert test was applied in 60% of these cases, all of which yielded the right diagnosis. The measured HNP1-3 level was 5062 (4666–6224) pg/mL. Of note, three cases were classified as possible infections and were admitted one day after their first symptoms, a time point wherein the yield of the HNP1-3 test was proved marginal.

### 3.2. Baseline Data of the Whole Study Population According to Quartiles of HNP1-3

When testing for possible correlations between HNP1-3 levels and demographic parameters (Table 7), we found only age to be inversely related to HNP1-3 levels.

We then tested for associations between HNP1-3 and clinical parameters and biomarkers (Table 8). After performing multivariable linear regression analysis applying the Pearson correlation, we found positive correlations between HNP1-3 and neutrophils (*p* < 0.001, r = 0.41), WBC (*p* < 0.001, r = 0.4), and CRP (*p* = 0.018, r = 0.226), while finding a negative correlation with age (*p* = 0.029, r = −0.207).

We finally evaluated the value of the different categorical variables in predicting viral vs. bacterial infections (Table 9). Only age and CRP (at values >42.3) were significantly correlated with bacterial diagnosis prediction.

## 4. Discussion

Visits for fever at pediatric emergency departments have resulted in a huge clinical and economic burden on the health care system. In the USA alone, 180,000 pediatric ER visits for fever were reported between 2007 and 2017 for children aged ≤90 days, and 2.6 million visits were reported for children aged 91 days–2 years [24]. In a different USA report, 30 million pediatric ER visits were documented, 32% for infectious disease and 3.3% ending in hospitalization. Effective management of febrile children is mainly impeded by the clinical difficulty in differentiating between bacterial and viral infections [14]. This clinical ambiguity drives both antibiotic underuse and overuse [25] with negative implications for morbidity, mortality, and the emergence of antibiotic resistance.

Prompt differentiation between bacterial and viral illnesses and timely application of appropriate antibiotic/antiviral therapy are crucial for better patient prognosis/survival. Intuitively, a biomarker that can discriminate between bacterial and viral infections with reasonable sensitivity and specificity will enable expeditious and efficient pharmacotherapeutic management and improve clinical outcomes. Multiple biomarkers/factors serve as valuable tools in the differentiation between viral and bacterial agents including WBCs, neutrophils, platelets, neutrophil/lymphocyte ratio, CRP, and the recently introduced ImmunoXpert kit [26].

HNP1-3 are the major neutrophilic peptides. They are stored in the azurophilic granules of neutrophils and released upon activation by exocytosis. HNP1-3 are a fundamental part of the first line of defense in the innate immune system against infectious agents. Their hydrophobic structure enables their attachment to pathogens’ membranes, forming destructive pores that induce fluid efflux with subsequent lysis. In healthy individuals, normal plasma HNP1-3 levels are found in the nanomolar range (<15 nM), with a marked elevation during acute inflammatory insults, primarily acute bacterial infections (as high as 50 μM) [27]. Successive studies indicated a clear association between bacterial infection and neutrophil stimulation as well as between viral infection and lymphocytosis [28,29,30]. Moreover, the human neutrophil lipocalin, which is primarily a neutrophil secretory protein, was found to be helpful in discriminating acute viral from bacterial infections with positive and negative predictive values >90% [31]. Since HNP1-3 are the major neutrophilic peptides intimately linked to pathogen destruction, and as previously shown considering the diagnostic value of the neutrophilic protein lipocalin, we sought in the current paper to delineate possible associations between HNP1-3 levels and bacterial, rather than viral, infection.

We found substantially higher HNP1-3 levels in patients with bacterial diagnoses, primarily in definitive and probable cases. The predictive value of the HNP1-3 test was higher in cases where the patients presented between 2 and 6 days from the first symptoms’ onset. Impressively, using a threshold value of 8500 pg/mL, we could truly recognize 70% of the antibiotic-misuse cases which had a definite or probable diagnosis. Overall, the accuracy of the HNP1-3 test at the latter cutoff was 73%, and it was even higher (86%) when excluding early (less than 1 day) or late (more than 6 days) admissions from disease onsets. The ImmunoXpert assay was used in 40% of the later cases, yielding a correct diagnosis in only 40%. We also found a negative correlation between HNP1-3 levels and patient age. In the COVID-19 era, it was shown that children have a stronger innate immune response in the nasopharyngeal mucosa than do adult patients [32], which could partially explain the lower susceptibility to infection at younger ages with a more “developed” innate response. HNP1-3 are considered to be the first line of innate defense against infections, which can potentially explain why HNP1-3 levels are higher at younger ages where the adaptive immunity is still immature. Moreover, vitamin D was proved as a positive modulator of HNP1-3 expression [33], and the prevalence of vitamin D deficiency was found to be higher among older children, again potentially explaining lower HNP1-3 levels in older children [34]. Altogether, higher HNP1-3 levels in younger children might be ascribed to both higher vitamin D levels and less developed adaptive immune systems, giving rise to robust innate immunity in the first years of life regardless of the pathogenic agent involved.

Despite the above correlations, HNP1-3 failed in multiple regression analysis to exhibit a significant predictive yield in discriminating between viral and bacterial infections. In a similar manner, multiple bacterial-induced host proteins, including procalcitonin, C-reactive protein (CRP), and Interleukin-6, have been previously suggested to support the diagnosis of infection with limited precision [35,36,37,38,39]. Their performance was negatively impacted by inter-patient variability, including in the time from symptom onset, the severity of the clinical presentation, and the pathogens implicated, among other factors. Likewise, HNP1-3 are hydrophobic molecules that are cleared rapidly (t^1/2^ of 9.7 min) from the circulation without being excreted in the urine or feces [22]. We showed here a correlation between the timing of presentation from symptoms’ onset and variation in HNP1-3 levels. The latter were also negatively correlated with age and are reported to be negatively affected by baseline vitamin D levels [14]. Overall, we tested neutrophil response to infection quantitatively by cell counts and qualitatively by the measurement of HNP1-3 levels. The current results demonstrate that the quantitative assessment/neutrophil number is stable and more predictive and pathogen-specific than the fluctuating qualitative variable, namely HNP1-3.

## 5. Conclusions

The plasma HNP1-3 test is an accessible and convenient tool. The measurement of HNP1-3 levels seems diagnostically supportive in patients with febrile illness of likely bacterial origin, especially if the duration of symptoms is more than 24 h. Successive studies demonstrated distinctive time dynamics of different blood biomarkers. Judicious implementation of the HNP1-3 test might prove helpful. Nevertheless, further larger studies are needed to establish HNP1-3 utility.

## 6. Study Limitation

The current study evaluated children at a mean age of 6.3 years from a Middle Eastern population throughout a distinct period of the year. The generalizability of the results necessitates testing larger numbers of patients during different months/seasons of the year as well as better testing of other ethnical groups. We also did not address variables potentially affecting HNP1-3 levels, including vitamin D levels. Finally, the number of participants was relatively low, with only 45% labeled as having a definitive diagnosis. A larger study recruiting a more restricted age group, using a clearer and shorter time interval from symptom onset to enrollment, and assessing only definitive diagnosis patients is mandatory to shed light on the predictive diagnostic role of HNP1-3.

## Figures and Tables

**Table 1:** Patient demographic and clinical markers at admission according to the study group.

	Control	Bacterial	Viral
N	13	41	57
Female:Male	5:8	19:22	26:31
Age	8.2 ± 5	5.8 ± 4.8	4.5 ± 4.5
Weight	32.6 ± 16.4	26.7 ± 22.4	18.2 ± 12.9
Fever	36.8 ± 0.4	38.4 ± 1.1	38.4 ± 1.2
Sat%	99.2 ± 1%	97.7 ± 2.1%	97.9 ± 1.9%
Heart rate	93.5 ± 21.4	138.3 ± 26.9	139.4 ± 28.8

**Table 2:** Discharge diagnosis categories in both groups. UTI—urinary tract infection, URTI—upper respiratory tract infection.

Bacterial (n)	Viral (n)
Pneumonia (22)	URTI (36)
UTI (5)	Gastroenteritis (8)
Gastroenteritis (4)	Pneumonia (8)
URTI (5)	Nondetermined (5)
Otitis media (4)	
Osteomyelitis (1)	

**Table 3:** Patient diagnosis classification in the two groups as given by the expert panel.

Bacterial (n)	Viral (n)
Definite (13)	Definite (31)
Probable (22)	Probable (0)
Possible (6)	Possible (26)

**Table 4:** Isolated pathogens among the definite diagnosis subgroups in both groups. RSV—respiratory syncytial virus, EBV—Epstein–Barr virus.

Definite Viral (n)	Definite Bacterial (n)
*H. influenza* A (17)	*E. coli* (4)
*H. influenza* B (2)	Shigella (2)
Adenovirus (5)	*H. influenza* (2)
RSV (4)	Streptococcus A (2)
EBV (3)	MRSA (2)
	Campylobacter (1)

**Table 5:** Different biochemical and hematological determinants in both groups. CRP—c-reactive protein, WBC—white blood cells.

	Bacterial; n = 41	Viral; n = 57	*p* Value
HNP1-3	10,428 [5789–14,866]	7352 [3762–10,672]	*p* = 0.007
CRP	107 [45–215]	20.5 [4.6–42.4]	*p* < 0.001
WBC	17.2 [10.2–23.1]	8.9 [6.7–14.85]	*p* < 0.001
Neutrophils	11.5 [6.3–17.8]	5.15 [2.15–8.75]	*p* < 0.001
Lymphocytes	2.67 [1.40–5.56]	3.13 [1.54–4.94]	*p* = 0.90
Platelets	331 [238–416]	287 [310–397]	*p* = 0.22
Glucose	104.5 [96.3–117.8]	98 [90–104.5]	*p* = 0.005

**Table 6:** HNP1-3 levels in the different subgroups.

	Bacterial	Viral
Definite infection	10,546	6931
Probable infection	13,271	
Possible infection	10,545	10,672

**Table 7:** Correlations between HNP1-3 levels and different demographic parameters. BMI—body mass index.

HNP1-3 Tertiles [pg/mL]	156–3808 (1)	3808–7433 (2)	7433–10,998 (3)	>10,998 (4)	*p* Value
Age	8 [2.5–14.8]	2.5 [1.1–6.3]	3.5 [1.3–8.0]	2.15 [1.43–8.38]	p^1^ = 0.009 p^3^ = 0.056
Gender					p = 0.61
Male	18 (64%)	15 (54%)	15 (56%)	13 (46%)	
Female	10 (36%)	13 (46%)	12 (44%)	15 (54%)	
BMI	19.4 ± 5.6	18.4 ± 5.5	17.8 ± 2.85	17.1 ± 2.40	p = 0.85
Ethnicity					p = 0.89
Jewish	11 (39%)	12 (43%)	13 (48%)	11 (39%)	
Arab	17 (61%)	16 (57%)	14 (52%)	17 (61%)	

**Table 8:** Age and different blood tests according to HNP1-3 tertiles. p^1^ = 1 vs. 2, p^2^ = 1 vs. 3, p^3^ = 1 vs. 4, p^4^ = 2 vs. 3, p^5^ = 2 vs. 4.

HNP1-3 Tertiles [pg/mL]	156–3808 (1)	3808–7433 (2)	7433–10,998 (3)	>10,998 (4)	*p* Value
Age	8 [2.5–14.8]	2.5 [1.1–6.3]	3.5 [1.3–8.0]	2.15 1.43–8.38	p^1^ = 0.009 p^3^ = 0.056
CRP	2.25 [0.63–9.2]	26.2 [5.12–53.8]	41.4 [20.12–140.2]	92.6 [28.7–144.8]	p^1^ = 0.049 p^2,3^ < 0.001 p^4^ = 0.046
WBC	7.2 [5.05–8.6]	11.3 [6.8–14.0]	15.0 [9.2–21.0]	16.3 [9.5–21.4]	p^2,3^ < 0.001 p^5^ = 0.052
Neutrophils	3.2 [1.7–5.3]	5.6 [2.2–10.0]	8.6 [4.1–15.4]	10.8 [6.3–15.5]	p^2,3^ < 0.001 p^5^ = 0.007
Lymphocytes	2.3 [1.7–3.4]	2.7 [1.5–5.4]	3.4 [2.34–5.6]	3.16 [1.35–5.4]	p = 0.27
Platelets	255.5 [210–336]	3074 [204–401]	322 [264–369]	316 [215–445]	p = 0.16
Glucose	96 [90–105]	99 [89–102]	104 [93–118]	103 [97–118]	p = 0.11

**Table 9:** Logistic regression analysis of continuous parameters to predict the diagnosis of bacterial infection. CRP—c-reactive protein, WBC—white blood cells.

		B	*p* Value	Odds Ratio	95% CI. for EXP(B)	
					Lower	Upper
Step 1	n_HNP1-3 8027 (1)	−0.036	0.953	0.964	0.289	3.219
	n_CRP.42.3(1)	1.805	0.004	6.081	1.766	20.937
	n_WBC.12.4(1)	−0.001	0.999	0.999	0.123	8.091
	n_Neutrophils (1)	1.424	0.142	4.154	0.621	27.785
	Platelets	0.000	0.843	1.000	0.995	1.004
	Lymphocytes	0.105	0.389	1.111	0.874	1.412
	Age	0.170	0.013	1.185	1.037	1.354
	Gender (1)	−0.424	0.451	0.654	0.217	1.970
	Constant	−2.717	0.008	0.066		

## Data Availability

This study is based on real-world patient data, including demographics and comorbidity factors, that cannot be communicated due to patient privacy concerns.
